# Application of quinpirole in the paraventricular thalamus facilitates emergence from isoflurane anesthesia in mice

**DOI:** 10.1002/brb3.1903

**Published:** 2020-10-30

**Authors:** Yawen Ao, Bo Yang, Caiju Zhang, Sirui Li, Haibo Xu

**Affiliations:** ^1^ Department of Radiology Zhongnan Hospital of Wuhan University, Wuhan University Wuhan China

**Keywords:** electroencephalogram, emergence, isoflurane anesthesia, paraventricular thalamus, quinpirole

## Abstract

**Background and Purpose:**

Dopamine is well‐known to contribute to emergence from anesthesia. Previous studies have demonstrated that the paraventricular thalamus (PVT) in the midline nuclei is crucial for wakefulness. Moreover, the PVT receives dopaminergic projections from the brainstem. Therefore, we hypothesize that the dopaminergic signaling in the PVT plays a role in emergence from isoflurane anesthesia.

**Methods:**

We used c‐Fos immunohistochemistry to reveal the activity of PVT neurons in three groups: The first group (iso^+^EM^‐^) underwent the anesthesia protocol and was sacrificed before emergence. The second group (iso^+^EM^+^) underwent passive emergence from the same anesthesia protocol. The last group (oxy^+^) received oxygen. D2‐like agonist quinpirole (2 or 4 mM) or D2‐like antagonist raclopride (2 or 5 mM) was microinjected into the PVT, and their effects on emergence and induction time were analyzed. Surface cortical electroencephalogram (EEG) recordings were used to explore the effects of quinpirole injection into the PVT on cortical excitability during isoflurane anesthesia. The activity of PVT neurons after quinpirole injection was assessed by c‐Fos immunohistochemistry.

**Results:**

The number of c‐Fos‐positive nuclei for the iso^+^EM^+^ group was significantly higher than the oxy^+^ and iso^+^EM^‐^ groups. Application of quinpirole (4 mM) into the PVT shortened emergence time compared with the saline group (*p* < .01). In contrast, administration of raclopride (2 mM) delayed emergence time (*p* < .05). Neither quinpirole nor raclopride exerted an effect on induction time. EEG analyses showed that quinpirole (4 mM) decreased the burst suppression ratio during isoflurane anesthesia (*p* < .01). The number of c‐Fos‐positive nuclei for the quinpirole (4 mM) group was significantly higher than saline group (*p* < .01).

**Conclusions:**

Our findings suggest that the activity of PVT neurons is enhanced after emergence from anesthesia, and the dopaminergic signaling in the PVT may facilitate emergence from isoflurane anesthesia.

## Graphical abstract

The activity of PVT neurons is enhanced after emergence from isoflurane anesthesia in mice. The dopaminergic signaling in the PVT facilitates emergence from isoflurane anesthesia.

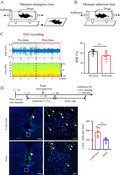

## 1 INTRODUCTION

Emergence from general anesthesia is clinically treated as a passive process whereby the general anesthetic is merely discontinued at the end of surgery, and recovery of consciousness is governed by anesthetic drugs to wear off. Drugs that accelerate recovery from general anesthesia may be useful for the treatment of emergence delirium, delayed emergence, and postoperative cognitive dysfunction (Solt et al., 2014). The pharmacological activation of cholinergic (Alkire et al., 2007), histaminergic (Luo & Leung, 2009), orexinergic (Zhang et al., 2012), and noradrenergic pathways (Fu et al., 2017) elicits arousal responses during general anesthesia. With respect to other monoamine system, dopamine (DA) is also well‐known to elicit wakefulness (Monti & Monti, 2007) and is thus of particular interest when considering arousal responses or emergence from general anesthesia. Electrolytic lesions of dopaminergic neurons have been shown to induce a coma‐like state (Jones et al., 1973). In addition, DA‐deficient mice have marginal arousal and appear to be hypoactive and apathetic (Palmiter, 2011). In contrast, electrical stimulation or optogenetic activation of the major DA nuclei of the ventral tegmental area (VTA) induces reanimation during general anesthesia with isoflurane or propofol (Solt et al., 2014; Taylor et al., 2016). Collectively, this available evidence strongly suggests that DA plays a promotive role in arousal regulation.

Based on human neuroimaging studies, Alkire and colleagues have proposed the thalamic switch hypothesis identifying a reduction of thalamic metabolism and blood flow as a common feature of most anesthetics (Alkire et al., 2000). Furthermore, accumulating evidence suggests that the midline thalamic nuclei in the brain have been shown to promote emergence from general anesthesia. For example, central medial thalamus (CMT) is a component of the midline thalamic nuclei and pharmacological activation of nicotinic acetylcholine or noradrenergic receptors in the CMT both cause emergence from anesthesia in rats (Fu et al., 2017; Van der Werf et al., 2002). Similarly, the paraventricular thalamus (PVT) is an affective function related region located in the dorsal part of the midline thalamus (Vertes et al., 2015) and has been heavily implicated in regulating sleep/wake networks (Colavito et al., 2015). Recent studies have indicated that the PVT is a critical node for controlling wakefulness (Matyas et al., 2018; Ren et al., 2018). Of note, PVT receives many of the chemically characterized fiber systems innervation containing dopaminergic, noradrenergic, and serotonergic fibers from the brain stem, as well as histaminergic, orexinergic, and neurotensin‐containing fibers from the hypothalamus, which are part of the sleep/wake‐regulatory networks (Berridge et al., 2012; Brown et al., 2012; Pace‐Schott & Hobson, 2002; Ren et al., 2018). Several lines of evidence have indicated that PVT participates in emergence from isoflurane anesthesia, yet whether dopaminergic signaling in the PVT has a promotive role on emergence from isoflurane anesthesia has not been well defined.

Since DA plays an important role in behavioral arousal and the PVT receives dopaminergic projections from the brainstem, we hypothesized that the dopaminergic signaling in the PVT plays a role in emergence from isoflurane anesthesia. In the present study, we first assessed whether the PVT is involved in emergence from isoflurane anesthesia using the c‐Fos immunohistochemistry. We then investigated the effects of D2‐like agonist quinpirole and antagonist raclopride on induction time or emergence time. In addition, we also recorded surface EEG to assess electrophysiological changes induced by quinpirole or raclopride during continuous isoflurane anesthesia. Moreover, to evaluate the influence of quinpirole on the activity of PVT neurons, c‐Fos immunohistochemistry was performed after infusion of quinpirole in the PVT.

## 2 MATERIAL AND METHODS

### 2.1 Animals

Male C57BL/6J mice (25–30 g) were purchased from the Hubei Research Center of Laboratory Animals. The guidelines of the National Research Council Guide for the Care and Use of Laboratory Animals were strictly followed throughout the experiment. Only males were chosen to reduce variability due to endocrine cycling. All mice were given food and water ad libitum with a controlled temperature (22 ± 2°C) under a 12‐hr light/dark cycle with lights on at 07:00. Mice were acclimated to the animal facility at least 7 days before experiments. All tests were performed during the light phase. All efforts were made to minimize the number of mice used and their suffering.

### 2.2 Surgical procedures

Under pentobarbital sodium anesthesia (50 mg/kg, i.p.), mice were placed on a stereotaxic apparatus (Narishige). An incision was cut along the midline, and the surface of the skull was exposed. The skull was cleaned with 3% H_2_O_2_ and dried with sterile cotton swabs. A guide cannula (O.D., 0.48 mm; I.D., 0.34 mm; length, 3.5 mm; RWD, Inc.) was implanted into the PVT (anteroposterior (AP) −1.1 mm from bregma; mediolateral (ML) +0.55 mm from midline; dorsoventral (DV) −3.25 mm from skull surface with 10° angle toward the midline to avoid damage to the superior sagittal sinus), according to stereotactic coordinates from the mouse brain atlas. In addition, mice were implanted with two stainless screw electrodes (recording electrode: AP = 1.75 mm, ML = −0.4 mm and reference electrode: cerebellum). The EEG electrodes were affixed to the skull with Super‐Bond C&B and dental acrylic. Before removing from the stereotaxic apparatus, a dummy inner cannula (length: 3.5 mm) was inserted into the guide cannula and the screw cap was tightened. After surgery, mice were given subcutaneous injection of carprofen (5 mg/kg, diluted in saline) and kept on a heating pad until revival, and then, mice were housed individually to decrease the incidence of cases in which the skullcap was removed. All mice were allowed to recover for at least 1 week before the subsequent experiments.

### 2.3 Drug preparation and administration

(−)‐Quinpirole hydrochloride (Sigma‐Aldrich, CAS No: 85798–08–9) and S(−)‐Raclopride (+)‐tartrate salt (Sigma‐Aldrich, CAS No: 98185–20–7) were dissolved in sterile saline. Drug or saline was delivered through an intra‐PVT inserted infusion cannula (O.D., 0.30 mm; I.D., 0.14 mm; length, 4 mm; RWD, Inc.) and infused at a rate of 0.25 μl/min for 2 min (total injection volume: 0.5 μl) by a microinjection pump (KD Scientific Infusion Syringe Pumps). The infusion cannula was designed to protrude 0.50 mm from the tip of the guide cannula and thus penetrate into the PVT. Investigators were blind to the drugs administered.

### 2.4 Experiment protocol

#### 2.4.1 c‐Fos expression in the PVT after emergence from isoflurane anesthesia

**Figure 1:**
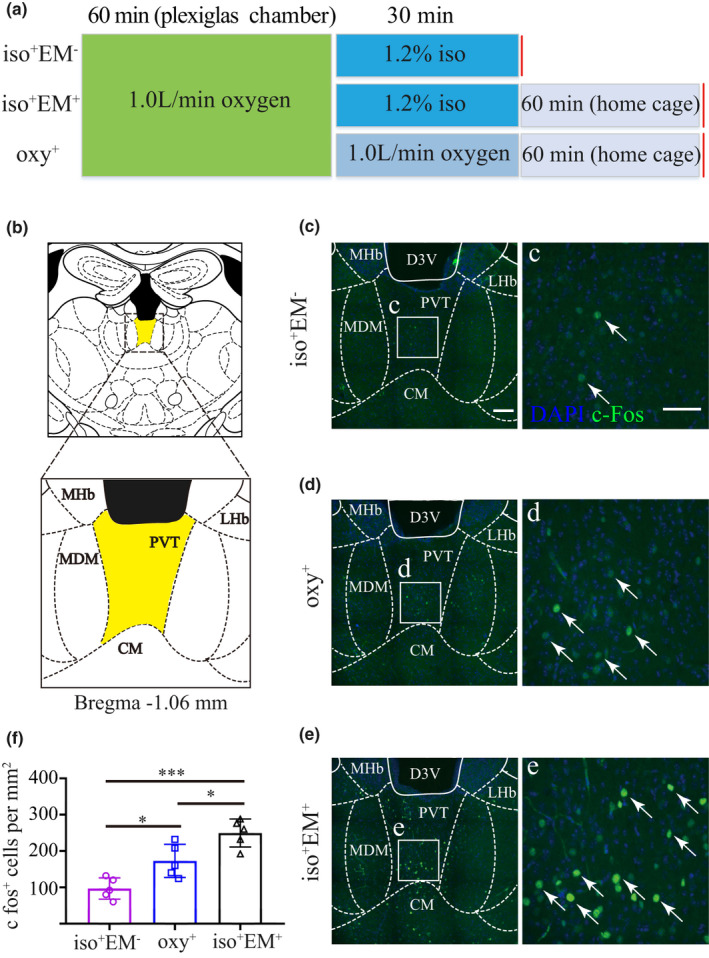
The PVT is active after emergence from isoflurane anesthesia. (a) A summary of the c‐Fos quantification experiments design scheme. The first group (iso^+^EM^‐^) underwent the isoflurane anesthesia protocol and was sacrificed before emergence. The second group (iso^+^EM^+^) underwent passive emergence from the same anesthesia protocol. The last group (oxy^+^) received only oxygen. (b) Image showing the location of the PVT (yellow area) and local structures in mouse. (c–e) Left: Representative coronal images showing c‐Fos expression in the PVT from the iso^+^EM^‐^ (c), oxy^+^ (d), and iso^+^EM^+^ mice (e). Right: Enlarged view of inset in the left panel showing c‐Fos expression in the PVT. (f) Statistical analysis of c‐Fos expression in the PVT from the iso^+^EM^‐^, oxy^+^, and iso^+^EM^+^ groups. Data shown as mean ± *SD* (*n* = 5, five slices per mouse). ^***^ *p* < .001, ^*^ *p* < .05. Data were analyzed by one‐way ANOVA with post hoc Bonferroni multiple‐comparison test. Scale bar: 250 µm in c, d, e (left panel), 100 µm in c, d, e (right panel)

It has been hypothesized that brain regions that control arousal become active after emergence and express c‐Fos. c‐Fos gene expression is rapidly induced in neurons after stimulation, with its protein product reaching a maximum 60–90 min after stimulation (Hoffman et al., 1993; Muindi et al., 2016). To identify the changes of the c‐Fos activity in the PVT after emergence from isoflurane anesthesia, fifteen adult male mice were randomly divided into three groups (*n* = 5 per group). Since the neurons in the PVT are robustly activated by unfamiliar stimuli and new environment (Zhou et al., 2018), mice were placed into the plexiglas chamber individually for at least 1 hr with 100% oxygen (1 L/min) flowing starting 3 days before the experiment. The time course of the c‐Fos expression experiment is schematized by Figure 1a. One group of mice was exposed to 30 min 1.2% anesthesia protocol but was sacrificed immediately at the end of the anesthesia procedure before emergence (iso^+^EM^‐^). Another group of mice was similarly exposed to 1.2% isoflurane for 30 min and then allowed to emerge (iso^+^EM^+^) in their home cage, 60 min after removed from the chamber the mice were sacrificed. The last group of mice was exposed only to 100% oxygen for 30 min and then sacrificed 60 min later (oxy^+^). The isoflurane concentrations were sampled from the distal end of the chamber using Drager Vamos Plus Anesthesia Monitor (Germany) and maintained constant through the study.

#### 2.4.2 Anesthesia induction or emergence test

To investigate the effect of D2‐like agonist quinpirole or antagonist raclopride on induction and emergence time, mice were numbered and randomly divided into five groups (*n* = 7 per group): quinpirole groups (2 or 4 mM), raclopride groups (2 or 5 mM), and saline group. We used two doses of quinpirole and raclopride based on previous study (Monti & Jantos, 2018). Quinpirole or raclopride was microinjected into the PVT 10 min before isoflurane infusion or 10 min before termination of isoflurane. The control group was microinjected with same volume of saline. Anesthesia was induced and maintained with 1.2% isoflurane in 100% oxygen at a flow rate of 1 L/min. Loss of righting reflex (LORR) was checked by turning the cage 90° every 15 s, a mouse did not turn itself prone onto all four limbs was considered LORR. The duration from onset of isoflurane exposure to LORR was recorded as induction time. Emergence time was recorded as the time at which a mouse righted itself (all four paws on the ground) from the time of removal from the isoflurane chamber.

#### 2.4.3 Surface electroencephalogram (EEG) recordings and analyses

To determine the burst suppression ratio (BSR) from the EEG following drug infusion, we administered 1.2% isoflurane to maintain the mice at an anesthetic plane, accompanied by steady burst suppression. EEG signals were amplified and collected by a RM‐6240EC device (Chengdu Instrument Factory) at a sampling rate of 800 Hz. The signals were filtered between 0.1 and 100 Hz. The BSR was calculated by using previously established methods (Vazey & Aston‐Jones, 2014; Vijn & Sneyd, 1998). Briefly, an EEG suppression was defined as an interval where the amplitude was within ± 15 μV for at least 100 ms. The BSR was calculated as the percentage of EEG suppression in a 3‐min interval before and after drug administration. Representative EEG waves and heat map EEG power spectrum in different groups of mice were done by using Matlab.

#### 2.4.4 c‐Fos expression in the PVT after quinpirole injection

To evaluate the influence of quinpirole on the activity of PVT neurons, c‐Fos immunohistochemistry was performed after application of quinpirole or saline in the PVT. After implantation of cannula, mice were divided into two groups (*n* = 5 per group) and were acclimated to the plexiglas chamber for 3 days before the experiment. On the day of the experiment, mice were adapted to the chamber for 10 min and anesthetized with 1.2% isoflurane. Thirty minutes later, mice received microinjection of 0.5 μl saline or quinpirole (4 mM) for 2 min. After anesthesia, mice were removed from the chamber and placed in home cage for 60 min before euthanatized for c‐Fos staining.

### 2.5 Tissue processing and immunohistochemistry

After behavioral tests, mice were deeply anesthetized with pentobarbital sodium and then transcardially perfused with ice cold 0.9% saline followed by 4% paraformaldehyde. Brains were first fixed in 4% paraformaldehyde overnight at 4°C, and then cut into 40 μm coronal sections on a vibratome (Leica VT1000S). For c‐Fos staining, brain sections containing PVT were chosen alternatively and incubated in 1 × PBS solution containing 0.3% Triton X‐100 and 10% normal goat serum (Bosterbio) for 2 hr. Then, sections were incubated with mouse monoclonal anti‐c‐Fos (1:1,000, Abcam Cat# ab208942, RRID: AB_2747772) overnight at 4°C. Then, sections were washed three times (10 min each) in 1 × PBS and then incubated in a secondary antibody, Alexa Flour 488‐labeled goat anti‐mouse antibody (1:600; Thermo Fisher Scientific Cat# A32723, RRID: AB_2633275) for 2 hr at room temperature. Following incubation with fluorescent secondary antibodies, sections were then washed by 1 × PBS solution three times (10 min each), mounted on glass microscope slides, dried, and coverslipped with mounting medium including 50% glycerol (Sigma‐Aldrich) and DAPI (5 μg/ml; Roche).

### 2.6 Examination of the injection site

After behavioral tests, the cannula sites of mice were verified. Mice were deeply anesthetized with pentobarbital sodium and then transcardially perfused with ice cold 0.9% saline followed by 4% paraformaldehyde. Brains were first fixed in 4% paraformaldehyde overnight at 4°C and then cut into 40 μm coronal sections on a vibratome. Brain sections were stained with DAPI for histological analysis of cannula sites. The location of the guide cannula was examined under an inverted fluorescence microscope according to mouse brain atlas (Paxinos & Franklin, 2004). If the position of the guide cannula was incorrect, the animal was excluded from the study.

### 2.7 Microscopy and quantification

Images were acquired with a PE spinning‐disk confocal microscope (UltraVIEW VoX; PerkinElmer). Quantification of c‐Fos within the PVT was performed as described previously (Ren et al., 2018). Briefly, the numbers of c‐Fos positive neurons in the PVT were counted at alternate sections from approximately Bregma −0.94 and to −1.46 mm (five sections per mouse) along the rostral‐caudal axis. The investigator blinded to the group information manually counted the c‐Fos‐positive cells in a ROI (a square area with a side length of 500 µ) located within PVT in each section using ImageJ (National Institutes of Health) software. A second blinded researcher confirmed the counting results. The number of positive cells per animal was expressed as the average number of five sections.

### 2.8 Statistical analysis

Data were presented as mean ± *SD*. Sample sizes were determined based on previous publications (Li et al., 2019; Muindi et al., 2016; Ren et al., 2018; Wang et al., 2019; Zhang et al., 2012). All statistical analyses were performed using the GraphPad Prism 7.0. Before analysis, all data were tested for normality using Shapiro–Wilk's test. One‐way ANOVA with post hoc Bonferroni multiple‐comparison test or unpaired *t* test was used in current research. The level of significance was set at *p* < .05.

## 3 RESULTS

### 3.1 Passive emergence from isoflurane anesthesia induced an enhanced c‐Fos expression in the PVT

To investigate the activity of PVT in the process of emergence from isoflurane anesthesia, the c‐Fos expression in the PVT was assessed under different protocols. Figure 1a shows the experiment protocol. Figure 1b shows the location of the PVT and local structures. Representative c‐Fos expression is shown in Figure 1c for mice that were sacrificed before emergence (iso^+^EM^‐^), in Figure 1d for mice that were exposed to oxygen (oxy^+^), and in Figure 1e for mice that passively emerged from isoflurane anesthesia (iso^+^EM^+^). Enlarged view in the right panel shows that a relative paucity of c‐Fos‐positive nuclei in iso^+^EM^‐^ mice (Figure 1c), a few c‐Fos positive nuclei in oxy^+^ mice (Figure 1d), and a large cluster of c‐Fos positive nuclei in iso^+^EM^+^ mice (Figure 1e). The counts of c‐Fos positive nuclei in the PVT for three distinct experimental protocols are shown in Figure 1f. The mean number of c‐Fos positive nuclei per mm^2^ was 96.8 ± 29.3 for the iso^+^EM^‐^ group, 172.8 ± 45.7 for the oxy^+^ group, and 249.6 ± 38.4 for the iso^+^EM^+^ group. The number of c‐Fos‐positive nuclei for the iso^+^EM^+^ group was significantly higher than the iso^+^EM^‐^ (*p* < .001) and oxy^+^ groups (*p* < .05). A significant difference was also detected between the iso^+^EM^‐^ group and oxy^+^ group (*p* < .05).

### 3.2 Microinjection of quinpirole facilitated emergence, but raclopride slightly delayed emergence

**Figure 2:**
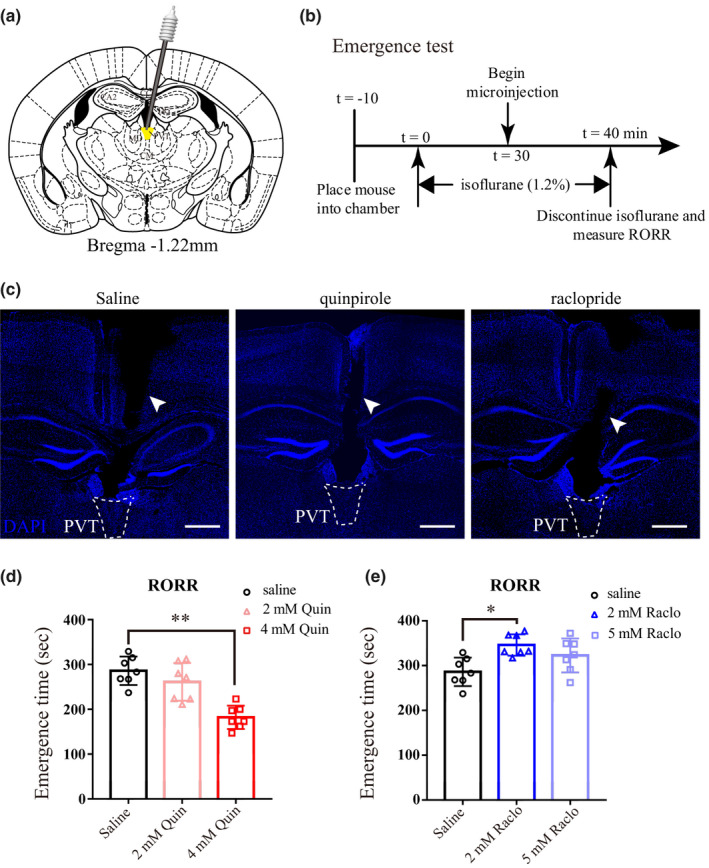
D2‐like agonist quinpirole in the PVT promotes emergence from isoflurane anesthesia. (a) Schematic representation of the implantation site of the guide cannula (black) above the PVT. The infusion cannula (gray) is within the PVT. (b) Pharmacological experiment protocol for emergence time. (c) DAPI stained coronal brain sections showing the track of the guide cannula (white arrow heads) above the PVT. Dashed white line indicates the outline of the PVT. (d and e) Scatter plot of emergence time. Microinjection of quinpirole (4 mM) facilitated emergence, in contrast, raclopride (2 mM) delayed emergence time. Data shown as mean ± *SD* (*n* = 7). **p < .01, *p < .05. Data were analyzed by one‐way ANOVA with post hoc Bonferroni multiple‐comparison test. Scale bar: 500 µm in c

Figure 2a shows schematic representation of the implantation site of the guide cannula above the PVT. Figure 2b shows the experimental protocol used to quantify the emergence time for isoflurane anesthesia. Figure 2c shows representative brain sections containing the track of the guide cannula. When compared with the saline control (286.0 ± 31.8 s), the emergence time was shortened with 4 mM quinpirole (182.1 ± 25.9 s versus. 286.0 ± 31.8 s, *p* < .01; Figure 2d), but not with the 2 mM quinpirole (261.3 ± 42 s versus. 286.0 ± 31.8 s, *p* > .05). Intra‐PVT injection of 2 mM raclopride slightly prolonged emergence time compared with the saline control (346.0 ± 23.8 s versus. 286.0 ± 31.8 s, *p* < .05; Figure 2e), while 5 mM raclopride showed no significant impact on emergence time (322.7 ± 37.74 s versus. 286.0 ± 31.8 s, *p* > .05; Figure 2e).

### 3.3 Neither quinpirole nor raclopride had an effect on the induction time

**Figure 3:**
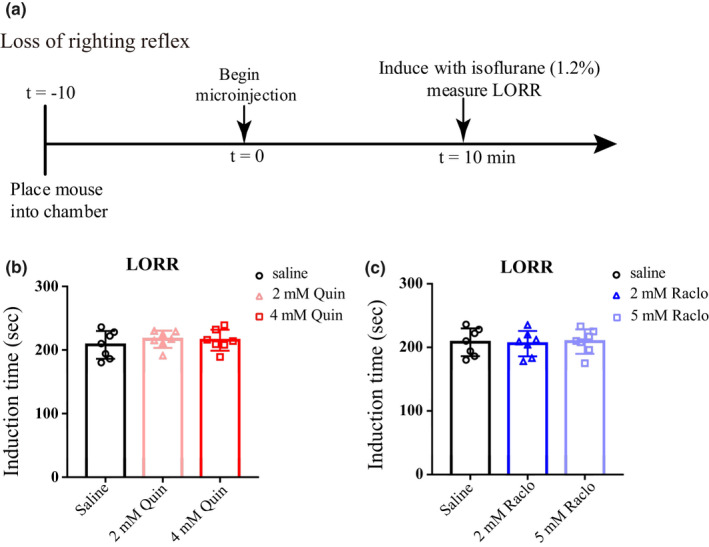
Neither quinpirole nor raclopride has an effect on the induction time. (a) Pharmacological experiment protocol for induction time. (b and c) Neither administration of quinpirole nor raclopride in the PVT exerted an effect on induction time. Data shown as mean ± *SD* (*n* = 7), *p* > .05. Data were analyzed by one‐way ANOVA with post hoc Bonferroni multiple‐comparison test

To determine the effect of quinpirole and raclopride on the induction time, we evaluated the loss of the righting reflex (LORR) after at least 3 days washout period. Figure 3a shows the experimental protocol used to quantify the induction time. Neither quinpirole nor raclopride altered the induction time compared with saline (*p* > .05; Figure 3b, c).

### 3.4 Microinjection of quinpirole into the PVT reduced BSR during isoflurane anesthesia

**Figure 4:**
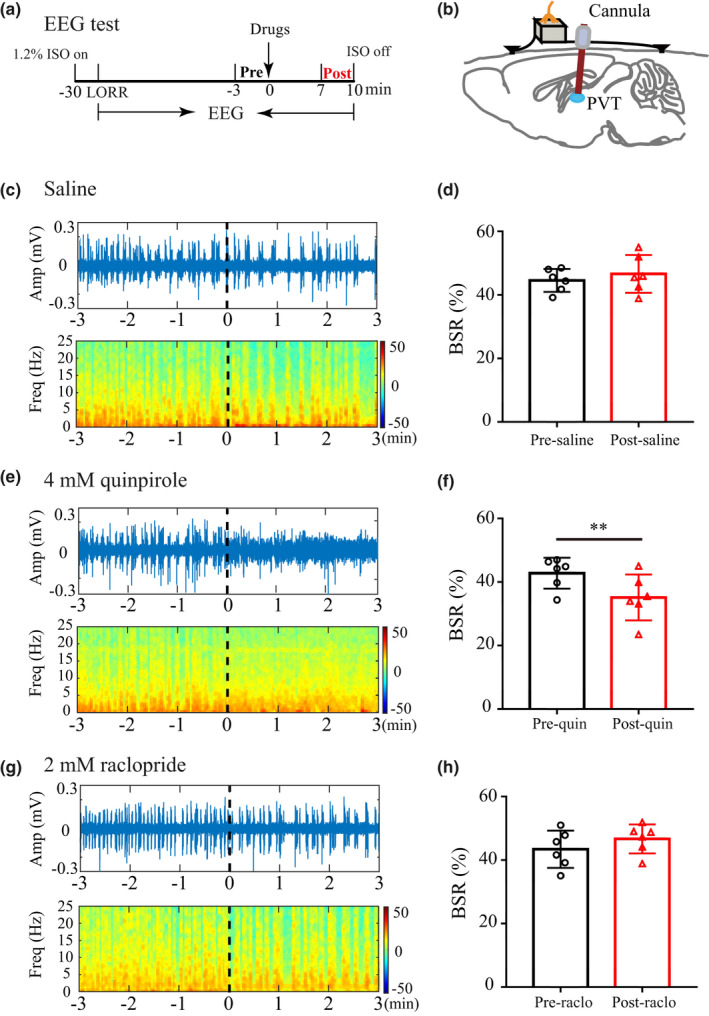
Quinpirole in the PVT reduces the BSR during isoflurane anesthesia. (a) Timeline for EEG recording in isoflurane anesthesia. EEG was recorded throughout the procedure, the 3‐min interval was used to compute BSR before administration (black, “Pre”) and after administration (red, “Post”). (b) Schematic diagram of guide cannula for drug administration and electrode for EEG recording. (c–d) Representative EEG waves and heat map EEG power spectrum before and after microinjection of saline, and saline did not induce significant changes in the BSR. (e–f) Representative EEG waves and heat map EEG power spectrum before and after microinjection of 4 mM quinpirole. Administration of quinpirole into the PVT decreased the BSR from 42.78% ± 4.83% to 35.15% ± 7.24% during 1.2% isoflurane anesthesia maintenance. (g–h) Representative EEG waves and heat map EEG power spectrum before and after microinjection of 2 mM raclopride. Raclopride induced no significant changes in the BSR. Data shown as mean ± *SD* (*n* = 6). **p < .01 for figure 4f, *p* > .05 for figure 4 d,h. Data were analyzed by paired *t* test

To assess electrophysiological changes induced by quinpirole or raclopride, we monitored EEG during continuous isoflurane anesthesia. The 3‐min interval was used to compute BSR before infusion and after infusion (Figure 4a). Schematic diagram indicated the location of guide cannula and EEG electrode (Figure 4b). As anesthesia progressed, burst suppression was observed (Figure 4c). Representative EEG waves and heat map EEG power spectrum in mouse microinjected with saline, 4 mM quinpirole or 2 mM raclopride were shown in Figure 4c, e, and g, respectively. No significant difference in average preinfusion BSR was observed between the saline, 4 mM quinpirole, and 2 mM raclopride groups. Application of 4 mM quinpirole into the PVT induced a significant decrease in BSR from 42.78% ± 4.83% to 35.15% ± 7.24% (*p* < .01; Figure 4f), whereas microinjection of saline or 2 mM raclopride into the PVT caused no significant changes in BSR (Figure 4d and h).

### 3.5 Administration of quinpirole increased the activity of PVT neurons

**Figure 5:**
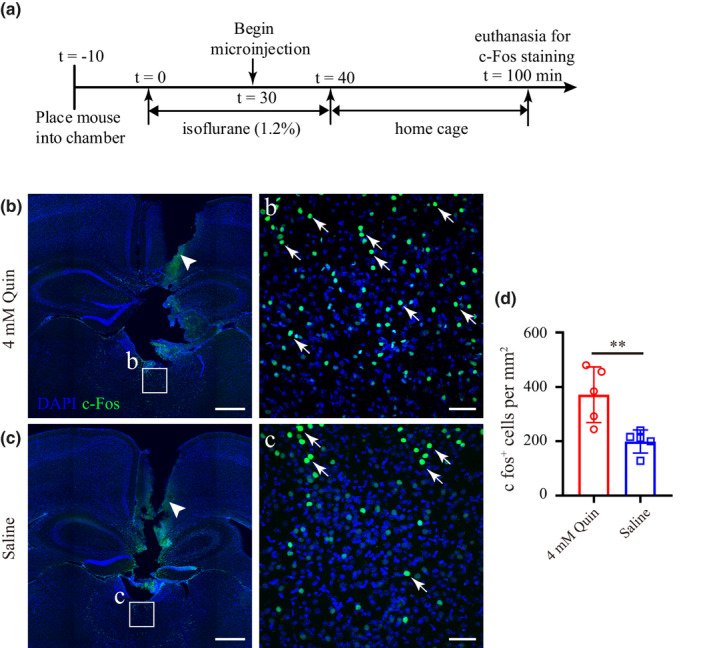
Quinpirole in the PVT increases the c‐Fos activity. (a) Experimental timeline for c‐Fos staining after quinpirole or saline microinjection. (b, c) Coronal brain sections showing track of guide cannula (white arrow head) above the PVT and c‐Fos expression in mice microinjected with quinpirole (b) or saline (c). Inset (b, c) shown at higher magnification. (d) Statistical analysis of c‐Fos expression in the PVT after quinpirole or saline application. Data shown as mean ± *SD* (*n* = 5, five slices per mouse). **p < .01. Data were analyzed by unpaired *t* test. Scale bar: 500 µm in b and c, 50 µm in b and c

Giving the findings above, we investigated whether injection of quinpirole (4 mM) affected the activity of PVT neurons. Figure 5a shows the experimental protocol used to assess PVT activity after quinpirole or saline injection. The mean number of c‐Fos positive nuclei per mm^2^ was 371.2 ± 102 for the quinpirole group and 199.2 ± 42.4 for the saline group (Figure 5b–d). The number of c‐Fos‐positive nuclei in the quinpirole group was significantly higher than the saline group (*p* < .01).

## 4 DISCUSSION

In this study, we first found that the number of c‐Fos‐positive nuclei for the iso^+^EM^+^ group was significantly higher than the iso^+^EM^‐^ and oxy^+^ groups. At 1.2% isoflurane, administration of D2‐like agonist quinpirole (4 mM) into the PVT significantly shortened the emergence time and decreased the BSR from 42.78% ± 4.83% to 35.15% ± 7.24%. In contrast, D2‐like antagonist raclopride (2 mM) delayed emergence time and caused no significant change in BSR. In addition, neither quinpirole nor raclopride altered the induction time. Moreover, injection of quinpirole into the PVT significantly increased the number of c‐Fos‐positive nuclei. These results indicate that dopaminergic signaling in the PVT may accelerate emergence from isoflurane anesthesia, which has a potential to develop the treatment for shortening the recovery time from general anesthesia.

The PVT is a critical node in circadian timing, arousal, and sleep states (Colavito et al., 2015; Van der Werf et al., 2002). In the PVT, c‐Fos expression is higher after a period of wakefulness when compared to sleep in mice (Ren et al., 2018). Since c‐Fos is a static measure of cumulative activity of neurons (Vendrell et al., 1998), positive c‐Fos expression may indicate neural activity. In our work, c‐Fos expression in the iso^+^EM^−^ group was significantly lower than the oxy^+^ group. This inhibition in a wake‐promoting brain region during isoflurane agrees with previous reports where isoflurane inhibits c‐Fos activity in two other wake‐promoting area in the lateral hypothalamus and parabrachial nucleus (Kelz et al., 2008; Muindi et al., 2016). Moreover, c‐Fos expression in the iso^+^EM^+^ group was significantly higher than the oxy^+^ and iso^+^EM^−^ groups. Thus, these results suggest that the PVT plays an important role in passive emergence from isoflurane anesthesia.

DA modulates neural activity through the two major groups of DA receptors on the target neurons containing the D1‐like receptors (D1 and D5) and the D2‐like receptors (D2, D3, and D4) (Beaulieu et al., 2015). The role of the D2 receptor is much more complex than D1 receptor because it results from both presynaptic and postsynaptic expression of this type of receptor (Beaulieu & Gainetdinov, 2011; Missale et al., 1998). Genetically modified animal models and pharmacological manipulations support a role for D2 receptor in arousal regulation (Isaac & Berridge, 2003; Qu et al., 2008, 2010); thus, D2R knockout mice show decrease in wakefulness and intracerebroventricular injection of the D2R agonist promotes wakefulness. Based on the robust expression of D2 receptors in the PVT (Beas et al., 2018; Clark et al., 2017), we sought to investigate a potential involvement of dopaminergic signaling in the regulation of anesthesia wakefulness. In our study, the larger doses of quinpirole significantly promoted emergence from isoflurane anesthesia. Similarly, it has been shown that microinjection of larger doses of quinpirole into the dorsal raphe nucleus (DRN) significantly increases wakefulness compared with lower doses (Monti & Jantos, 2018). In addition, microinjection of raclopride into the PVT delayed emergence time, which again suggested that the dopaminergic signaling in the PVT could regulate the arousal. Because the D2 receptors are found both pre‐ and postsynaptically and have multiple effects on arousal, it has been hypothesized that the increase in wakefulness after larger doses depends on activation of the postsynaptic D2 receptors (Monti & Monti, 2007). More recently, it has been shown that dopaminergic signaling increases neuronal excitability within the PVT through activation of D2Rs (Beas et al., 2018; Gao et al., 2020). It is likely that the role of the PVT in promoting wakefulness could be associated to the dopaminergic signaling. In line with this, we observed a significant increase in PVT c‐Fos activity after quinpirole injection. It should be noted that D2R‐mediated signaling is diverse. D2Rs have classically been assumed to inhibit neuronal excitability via Gi signaling (Bonci & Hopf, 2005). However, D2R can modulate intracellular calcium levels by acting on ion channels or by triggering the release of intracellular calcium stores (Beaulieu & Gainetdinov, 2011). In addition, it has been shown that D2Rs can enhance the excitability of pyramidal neurons in layer five of prefrontal cortex through pathways associated with Gs‐mediated signaling (Robinson & Sohal, 2017). Thus, quinpirole may participate in accelerating emergence from isoflurane anesthesia via activation of PVT.

According to previous research, it has been hypothesized that the neural substrates governing transitions into and out of the anesthetized state may not be identical, so that induction and emergence from anesthesia are not a simple inverse process (Kelz et al., 2008). Our work showed that pharmacological manipulation of the PVT with D2‐like agonist quinpirole or antagonist raclopride did not change the induction time. Indeed, Fu et al., (2017) found that norepinephrine infusion into the central medial thalamic nucleus did not alter the induction time with propofol, but did accelerate emergence from anesthesia. Similar effects were noted that neither orexins nor orexin antagonists injected into the rat DRN exerted an impact on induction time (Yang et al., 2019). Thus, our work adds to the growing body of evidence that emergence from anesthesia is not a mere reflection of induction. However, it is still need to determine whether manipulation of specific neuronal groups in the PVT could affect induction and emergence from anesthesia.

Electroencephalogram is a continuous, noninvasive method that has been used as a measure of anesthetics action on the central nervous system (Rampil, 1998). Various anesthetics such as isoflurane, propofol, and barbiturates can induce burst suppression, and burst suppression is associated with a state of cortical hyperexcitability (Kroeger & Amzica, 2007). A widely used method for quantifying burst suppression is the burst suppression ratio (BSR) (Rampil et al., 1988). A significant reduction in BSR in cortical EEG, an indicator of anesthetic depth, indicating a transition away from deep anesthesia (Vijn & Sneyd, 1998). In our study, application of 4 mM quinpirole dramatically reduced the BSR and promoted emergence from isoflurane anesthesia. However, application of 2 mM raclopride induced no significant changes in BSR. Similar findings have been reported that microinjection of orexin‐A in the VTA elicits a reduction in BSR and facilitates emergence from isoflurane anesthesia, while infusion of orexin receptor antagonists does not affect BSR (Li et al., 2019). In our study, microinjection of quinpirole in the PVT elicited a significant increase in c‐Fos expression. Therefore, we speculate that this arousal effect may be attributed to activation of PVT by quinpirole.

The current study has several limitations. First, future studies are required to clarify the types of neurons in the PVT involved in this process. Second, administration of quinpirole into the PVT cannot entirely mimic the effects of endogenous dopamine. Importantly, quinpirole and raclopride have high affinity for both D2R and D3R (Seeman & Schaus, 1991; Seeman & Van Tol, 1994). There is also a weak expression of D3R in mice PVT (Beas et al., 2018). Thus, pharmacological studies alone could not rule out the possibility that D3R contributes to the quinpirole‐induced c‐Fos expression in PVT or accelerated emergence. However, based on previous studies and a robust expression of D2R in the PVT (Beas et al., 2018), the effect we observed in this study may mainly attributed to D2R in the PVT.

## 5 CONCLUSION

Our findings suggest that activation of dopaminergic signaling in the PVT facilitates emergence from isoflurane anesthesia. Furthermore, blockade of dopaminergic signaling in PVT delays emergence from isoflurane anesthesia. These findings sustain the notion that the dopaminergic system contributes to emergence from anesthesia.

## CONFLICT OF INTEREST

All authors declare no conflicts of interest.

## AUTHOR CONTRIBUTIONS

Y.W.A and H.B.X designed the experiments. Y.W.A, B.Y., and C.J.Z. performed the experiments. Y.W.A, C.J.Z., B.Y., and S.R.L collected and analyzed the data. Y.W.A and H.B.X wrote the manuscript. H.B.X supervised the project. All authors reviewed the manuscript.

### Peer Review

The peer review history for this article is available at https://publons.com/publon/10.1002/brb3.1903.

## Data Availability

Further information and requests for resources and reagents should be directed to and are available without restriction from the lead contact responsible for materials availability, Haibo Xu (xuhaibo1120@hotmail.com).
